# Supplementary material for: Subjective Perceptions of Occupational Fatigue in Community Pharmacists
Source: Pharmacy (Basel). 2023 May 9;11(3):84. doi: 10.3390/pharmacy11030084 (PMC10204370; doi:10.3390/pharmacy11030084)
Supplement: Supplementary file 1 [file pharmacy-11-00084-s001.zip › pharmacy-2356904-supplementary.pdf]

Supplementary Materials
File S1: Pharmacist Fatigue Instrument

Instructions: When answering the next set of questions, think back over the days you worked the last two weeks (14 days).

Using the calendar below, please indicate the dates and times that you worked during the last two weeks.

Sunday	Monday	Tuesday	Wednesday	Thursday	Friday	Saturday
March 6<br>*Example:*<br>*9 AM – 3 PM*	7	8	9	10	11	12
13	14	15	16	17	18	19
20	21	22	23	24	25	26
27	28	29	30	31		

1. **Thinking about the last two weeks… how many days did you work?**

   [ ] days

2. **Thinking about the last two weeks… how many hours did you work most days?**

   [ ] hours

3. **During the last two weeks, did you ever take a scheduled break during the workday? A scheduled break includes any paid or un-paid periods of time where you were not working, such as a lunch break.**

   ○ Yes
   ○ No

Instructions: Sometimes, pharmacists say that they feel different at various times of the workday, both physically and mentally. Think back over the days that you worked the last two weeks. When answering the questions think of the pharmacy you work in the most. To answer the questions, please mark the appropriate response on the scale.

4. Think back over the days that you worked during the last two weeks when answering these questions. On how many workdays did you …

	**None of the days you worked**	**Less than half of the days you worked**	**Half of the days you worked**	**More than half of the days you worked**	**All of the days you worked**
**a.** Feel that your energy decreased over the course of the day?	○	○	○	○	○
**b.** Feel more tired later in the day than you did at the beginning of the day?	○	○	○	○	○
**c.** Feel pain or discomfort anywhere in your body?	○	○	○	○	○
**d.** Have trouble thinking clearly at work, even for a short time?	○	○	○	○	○
**e.** Have times where you forgot whether or not you had completed a task?	○	○	○	○	○
**f.** Have times where you spent longer completing a task later in the day than you would at the beginning of the day?	○	○	○	○	○
**g.** Have times where you felt that you could not keep up with your work?	○	○	○	○	○
**h.** Have times where you felt more impatient later in the day than you did at the beginning of the day?	○	○	○	○	○
**i.** Have times where you felt that you were not performing at your best?	○	○	○	○	○
**j.** Find it necessary to take short-cuts when providing patient care?	○	○	○	○	○
**k.** Have times where you felt that you were not able to go above and beyond standard patient care?	○	○	○	○	○
**l.** Have times where you felt generally fatigued?	○	○	○	○	○

5. Consider the last two weeks. At the end of most workdays, how tired did you feel?

- ○ Not at all tired
- ○ A little tired
- ○ Somewhat tired
- ○ Very tired
- ○ Extremely tired

Instructions: The next few questions ask about your thoughts on pharmacist fatigue **in general**, not just about your personal experiences.

6. Consider your thoughts in general, not just your personal experiences.

	Not at all	A little	Some	Quite a bit	A great deal
**a.** In general, how much do you think pharmacist fatigue affects patient safety?	○	○	○	○	○
**b.** In general, how concerned are you about fatigue in pharmacists?	○	○	○	○	○
**c.** How much do you think scheduled breaks during the workday would help pharmacists to cope with fatigue?	○	○	○	○	○

Thank you so much for taking the time to complete this survey.
Your thoughts and opinions are extremely important to us!

Table S1: Qualitative Codebook

Name	Description
Coping Mechanisms	Methods pharmacist list as helping to "cope" with or deal with feelings of occupational fatigue
Distal Outcomes	
Organization	Quality, Retention & Turnover, Profit
Patient	Patient Safety, Experience
Person	Burnout, Wellbeing, Health, Safety
Occupational Fatigue	
Active Fatigue	Overstimulation, High Cognitive Work, Overload, Continuous and Prolonged Demands
Passive Fatigue	Under stimulation, Low Cognitive Work, Underload
Mental Fatigue	Lack of Motivation, Sleepiness, Trouble Concentrating or "Fuzzy" Feeling when Working
Physical Fatigue	Physical Exertion, Physical Discomfort, General Lack of Energy
Structure & Process	
External Environment	Factors that the pharmacy or pharmacy organization are unable to change (e.g., insurance payers, COVID-19 pandemic, policy)
Organization	Teamwork, Culture, Policies, and Procedures, Training, Staffing
Person	Experience and Training, Knowledge, Personal Demographics or External Activities
Physical Environment	Physical Layout, Noise, Lighting, Temperature
Tasks	Activities that pharmacists complete as part of their daily work (e.g., checking prescriptions, administering vaccines, counseling)
Tools & Technology	Resources available to the pharmacist to help complete tasks and meet work demands (e.g., pharmacy dispensing system)
